# CT-based finite element simulating spatial bone damage accumulation predicts metastatic human vertebrae strength and stiffness

**DOI:** 10.3389/fbioe.2024.1424553

**Published:** 2024-07-23

**Authors:** Zahra Soltani, Michelle Xu, Raul Radovitzky, Marc A. Stadelmann, David Hackney, Ron N. Alkalay

**Affiliations:** ^1^ Department of Orthopedic Surgery, Center for Advanced Orthopedic Studies, Beth Israel Deaconess Medical Center and Harvard Medical School, Boston, MA, United States; ^2^ Institute for Soldier Nanotechnologies Aeronautics and Astronautics, Massachusetts Institute of Technology, Cambridge, MA, United States; ^3^ Department of Aeronautics and Astronautics, Institute for Soldier Nanotechnologies, Massachusetts Institute of Technology, Cambridge, MA, United States; ^4^ Centre for Artificial Intelligence, ZHAW School of Engineering, Zurich University of Applied Sciences, Zurich, Switzerland; ^5^ Department of Radiology, Beth Israel Deaconess Medical Center and Harvard Medical School, Boston, MA, United States

**Keywords:** spine, human metastatic vertebrae, finite element framework, bone damage model, fracture prediction

## Abstract

**Introduction:** Pathologic vertebral fractures are devastating for patients with spinal metastases. However, the mechanical process underlying these fractures is poorly understood, limiting physician’s ability to predict which vertebral bodies will fail.

**Method:** Here, we show the development of a damage-based finite element framework producing highly reliable pathologic vertebral strength and stiffness predictions from X-Ray computed tomography (CT) data. We evaluated the performance of specimen-specific material calibration vs. global material calibration across osteosclerotic, osteolytic, and mixed lesion vertebrae that we derived using a machine learning approach.

**Results:** The FE framework using global calibration strongly predicted the pathologic vertebrae stiffness (*R* ^2^ = 0.90, *p* < 0.0001) and strength (*R* ^2^ = 0.83, *p* = 0.0002) despite the remarkable variance in the pathologic bone structure and density. Specimen-specific calibration produced a near-perfect prediction of both stiffness and strength (*R* ^2^ = 0.99, *p* < 0.0001, for both), validating the FE approach. The FE damage-based simulations highlighted the differences in the pattern of spatial damage evolution between osteosclerotic and osteolytic vertebral bodies.

**Discussion:** With failure, the FE simulation suggested a common damage evolution pathway progressing largely localized to the low bone modulus regions within the vertebral volume. Applying this FE approach may allow us to predict the onset and anatomical location of vertebral failure, which is critical for developing image-based diagnostics of impending pathologic vertebral fractures.

## 1 Introduction

In 2022, more than 1.9 million new cancer cases were estimated in the US (Rebecca et al., 2022). Spinal bone metastases affect more than 70% of patients with advanced cancer (Cronin et al., 2018). Nearly half of the spinal metastasis patients undergo vertebral radiation therapy (RT) (Begg et al., 2011), with up to 40% of these patients suffering clinically significant vertebral fractures (VF) post-RT (van der Velden et al., 2017; Faruqi et al., 2018). Once VF occurs, patients may suffer severe impairment of quality of life (Pond et al., 2014), higher health costs (McDougall et al., 2016), and neurological deficits in up to 50% of patients with VF (Oster et al., 2013), shortening patient survival (Saad et al., 2007; Oster et al., 2013) and 3-year life expectancy (Oefelein et al., 2002; Pond et al., 2014). Clinical guidelines for predicting PV risk remain subjective with low accuracy VF (Yao et al., 2017). Establishing the risk of pathologic vertebral fractures before catastrophic pain or neurologic deficits occur remains an unmet, critical clinical need in managing patients with spinal metastatic disease.

The invasion of vertebral bone with bone metastases destroys vertebral anatomy (Taneichi et al., 1997). It disrupts the bone’s cellular homeostasis (Wu et al., 2018), causing remarkable degradation of the vertebral bone composition (Burke et al., 2016; Burke et al. 2017; Burke et al. 2017) and microarchitecture (Burke et al., 2016; Burke et al., 2017; Bailey et al., 2020; Bailey et al., 2022). Using a pre-clinical model of osteolytic and mixed vertebral bone tissue, (Atkins et al., 2019), showed this diminished bone quality to be associated with a higher accumulation of diffuse and linear microdamage and microfractures at regions of high stress when exposed to mechanical loading. These changes underly the well-documented worsening of the pathologic bone tissue’s mechanical properties (Nazarian et al., 2008; Burke et al., 2018; Bailey et al., 2020; Stadelmann et al., 2020).

Derived from CT data, computational finite element (FE) models allowed detailed insights into the effect of a range of osteolytic defects, simulated as idealized void-based geometries, on the failure strength of cadaver vertebra (Whyne et al., 2001; Tschirhart et al., 2004; Tschirhart et al. 2006; Tschirhart et al. 2007; Alkalay and Harrigan, 2016). In multiple myeloma patients with VF (Campbell et al., 2017) and in cancer patients with osteolytic vertebral lesions (Costa et al., 2019), subject-specific finite element (FE) highlighted the bone metastases’ deleterious effect on the patient’s vertebral mechanical competence, defined as lower vertebral stiffness and work to yield, as compared to cancer patients without observed vertebral fracture. Recent work employing digital image correlation (Palanca et al., 2023) demonstrated osteolytic lesions to cause significant alteration in the spatial strain distribution within the vertebrae and endplate, suggesting this effect as the mechanism for the observed vertebral failure. In vertebrae containing osteosclerotic lesions, Palanca et al. (2023) reported strain peaks within the vertebral volume to be largely focused around the osteosclerotic lesion and were associated with vertebral failure. However, the effect of osteosclerotic lesions on the mechanism of VF remains poorly understood.

A recent review of the CT-based finite element modeling for pathologic vertebrae highlighted the limitations of idealized and simplified lesion geometries and material models employed when developing patient-specific prediction of vertebral strength (Molinari and Falcinelli, 2022). Such development is crucial for developing clinical tools tailored to individualized treatments and diagnosis. Incorporating material characterization of osteolytic and osteosclerotic human bone,Stadelmann et al. (2020) compared a homogenized FE (hFE) model with that of a micro FE model for predicting the strength and stiffness of cadaveric vertebrae containing a wide range of osteolytic, osteosclerotic, and mixed bone metastases obtained from cancer patients. Although the study demonstrated the hFE approach utility for predicting the experimental strength, the authors did observe bone metastasis type, i.e., osteolytic vs. osteosclerotic vs. mixed (having both osteolytic vs. osteosclerotic within the vertebral volume), to markedly affect the FE models prediction accuracy for strength and stiffness. In total, these findings suggest that osteolytic and osteosclerotic lesions may differentially affect the process of damage evolution within the vertebral bone (Garcia et al., 2009) that affects vertebral failure (Jackman et al., 2016).

Bone is a multiscale, hierarchal biomaterial with complex mechanical responses at each scale (Schwiedrzik et al., 2013). A common approach for studying the effect of damage evolution on bone strength is to use FE simulations based on so-called continuum damage models, in which a single scalar variable *D* evolves irreversibly from 0 (no damage) to at most 1 (completely damaged, i.e., the material’s stiffness is fully diminished) according to a damage evolution law. Zysset and Curnier (1996) developed a general material model integrating isotropic damage and time-independent plastic flow, later expanded by Dall’Ara et al. (2013) with the central assumption that plastic flow and damage accumulation are intrinsically related. Incorporated into a three-dimensional continuum model of anisotropic trabecular bone, the model demonstrated strong correlations between predictions and experimental results for none-pathologic bone (Garcia et al., 2009; Wolfram et al., 2011) and vertebral strength (Chevalier et al., 2008; Dall’Ara et al., 2010). To describe a multi-axial yield and failure criterion for trabecular bone based on BV/TV, a scalar function of stress demarcates the boundary between the intact and damaged states of the bone. This function will consider the trabecular bone’s anisotropic and heterogeneous nature in the form of a halfspace generalization of the Hill criterion.

Alternative damage-based approaches employing elastic-plastic material failure (Molinari and Falcinelli, 2022) and phase field model (PFM) (Preve et al., 2024) have been proposed for investigating the failure of transpedicular screws in metastatic vertebrae. The former relies on a maximum stress criterion with the failed element assigned a negligible modulus to simulate failure. The PFM model relies on a critical energy release rate obtained using a power-law equality based on the bone density properties, assumed as locally soft for osteolytic bone and locally stiff for osteoblastic lesions with both lesion’s geometries modeled as spherical regions. In brittle, porous materials under compressive, the PFM model demonstrated the failure of load to be influenced by the size of the voids, with small voids promoting damage nucleation and enhancing the bridging of macro-pores through micro-crack formation (Cavuoto et al., 2024). In contrast, macro-pores affect the overall material response and drive the propagation of large fractures. These findings present a possible mechanism for the failure of osteolytic bone that does not demonstrate critical lytic foci but rather a “moth-eaten” presentation common to many such vertebrae (Bailey et al., 2022).

The study aims were to 1) extend the constitutive bone model proposed by Johnson et al. (2010), implemented in the ΣMIT FE framework (Radovitzky et al., 2011) to include a continuum bone damage computational framework. 2) Evaluate the new model’s accuracy for predicting the strength and stiffness of human vertebrae containing osteolytic osteosclerotic and mixed bone metastases 3) evaluate whether a single set of calibrated bone material parameters can be established to accurately predicts vertebral strength and stiffness independent of bone metastatic type. For this study, we simulated the measured strength and stiffness of ten cadaveric pathologic vertebrae containing osteolytic, osteosclerotic, and mixed bone metastases that were mechanically tested in a previous study (Stadelmann et al., 2020). We employed a machine learning approach to create a global material calibration scheme that would apply to all the bodies and tested the performance of specimen-specific vs. “global” material calibration on the model’s vertebral strength and stiffness prediction. We hypothesized that 1) the damage-based FE framework simulates the observed values for strength and stiffness and 2) a single set of model material constants provides a close simulation of the measured strength and stiffness across all bone metastasis types.

## 2 Materials and methods

### 2.1 Experimental measurements

For this study, we selected to simulate ten cadaveric vertebrae containing osteolytic, osteosclerotic or mixed bone metastases from a sample of 45 pathologic vertebrae that we mechanically tested in a previous study (Stadelmann et al., 2020). The vertebrae selected present a wide range of bone volume fraction values with either a high or a low measured strength or stiffness or a high difference in these mechanical properties. Hence, they represent the most challenging cases for modeling the measured experimental mechanical behavior.

#### 2.1.1 Specimens

Ten vertebral samples were obtained as part of our previous study (Stadelmann et al., 2020) from nine cadaver spines (three female, six male, age 49–71 years, mean age 54) from donors with solid cancer (three breast, three lung, two prostate, and one kidney), Table 1. As part of the previous study (Stadelmann et al., 2020), each spine was imaged in a clinical CT scanner, and upon radiographic review, vertebral segments exhibiting osteolytic, osteosclerotic, or mixed bone metastases were extracted. Following the protocol proposed by Dall’Ara et al. (2010), the vertebral end plates were sectioned to obtain plane-parallel segments, and the sectioned vertebrae prepared for micro CT scanning (Stadelmann et al., 2020).

**TABLE 1:** Demographic and physical properties of the vertebral specimens modeled.

Spine ID	Level	Cancer	Bone lesion	Age (Y)	Sex	Height (cm)	Weight (kg)	BMD (g/cm^3^)	Strength (kN)	Stiffness (kN/mm)
VA15-AL	T11	Prostate	S	71	M	188.0	68.0	250.01	8.52	19.75
MD15-L	L4	Lung	S	60	M	175.3	85.7	194.83	9.58	14.37
VA15-AL	T7	Prostate	S	71	M	188.0	68.0	146.66	4.31	12.6
VA15	L1	Breast	M	60	F	162.6	40.8	324.68	10.72	40.83
VA15-S	T11	Breast	S	60	F	162.5	40.8	318.3	11.12	36.84
AL15	L5	Breast	S	59	F	170.2	79.4	155.98	7.47	15.3
GA1	T11	Kidney	L	71	M	170.2	54.4	91.3	1.87	9.68
MD14	T9	Lung	L	49	M	177.8	68.0	124.89	3.42	11.82
PA15	T8	Breast	S	60	F	165.1	68.0	240.47	5.35	9.94
MD15-A	L1	Lung	S	60	M	182.9	81.6	186.05	5.83	14.86

Y: years; mgHA, milligram of hydroxyapatite crystal; Bone lesion: S, osteosclerotic; L, osteolytic; and M, mixed (osteolytic and osteosclerotic bone metastases within the vertebral volume).

#### 2.1.2 MicroCT imaging

Stadelmann et al. (2020): in brief, each vertebral section was imaged at 24.5 μm isotropic voxel size (μCT100, Scanco Medical, Switzerland) using the following parameters (tube voltage: 70 kV, tube current: 200 μA, integration time: 500 ms). A Gauss filter (Sigma: 0.8, Support: 1) (Chevalier et al., 2009) was applied to reduce high-frequency noise within the images, and the vertebra was segmented using a custom script (IPL, Scanco Medical, Switzerland). We computed the vertebral bone mineral density (BMC) for each image within the segmentation volume and, per image, applied an adaptive threshold algorithm to compute an optimal bone segmentation threshold (Ding et al., 1999; Burghardt et al., 2007). The mean threshold value (429 ± 56 mgHA/cm^3^) was applied to segment the bone tissue within the vertebral volume, and the resulting μCT data was used to derive the overall (i.e., cortical + trabecular regions) bone volume fraction (BV/TV).

#### 2.1.3 Mechanical experiment

Stadelmann et al. (2020): in brief, our study followed the standardized mechanical test protocol described by Dall’Ara et al. (2010). First, the specimen’s center of mass was computed from the *μ*CT images, digitally shifted anteriorly by a distance equal to 10% of the anteroposterior width of the vertebral bottom surface, and the image-derived location of force application and outer contour of the bottom surface printed on a sheet of paper to allow precise location of the specimen in the test system. This methodology aims to induce an anterior wedge-shaped fracture, a common fracture pattern in osteoporotic and cancer patients (Dall’Ara et al., 2010).

Each vertebral level was equilibrated in 0.9% NaCl saline solution for 1 h at room temperature, then placed and positioned based on the printed information on a fixed steel platen secured to the hydraulic testing system (858 Mini Bionix II, MTS, Eden Prairie, United States). With the specimen positioned, the cranial plate, having a ball joint mechanism to allow unconstrained deformation of the sample, was lowered until a tare load of 25 N was recorded to confirm contact. Both steel plates had their contact surfaces sandblasted to prevent the sample from sliding during the compression test. The vertebra was tested under monotonic uniaxial compressive displacement at a rate of 5 mm/min (Chevalier et al., 2009) until either a failure was registered or the built-in load cell (model: 662.20D-04) maximum force (15 kN) was reached. We defined vertebral strength (Fexp) as the maximum measured compressive force. Experimental stiffness was derived as the coefficient of the linear regression model fitted to 20%–80% of the load-displacement curve elastic region prior to the vertebral yield strength.

### 2.2 Establishing the damage-based FE framework

#### 2.2.1 Establishing elastic-plastic-damage model for vertebral bone

The model to simulate the pathologic vertebral bone elastic and plastic response was developed based on the constitutive law proposed by Johnson et al. (2010). Specifically, the modification incorporated a) bone volume fraction, $\left(\frac{BV}{TV}\right)$ , to capture the heterogeneity of bone volume density throughout the vertebrae and b) a three-dimensional elastic-plastic damage constitutive law for bone tissue (Garcia et al., 2009). The model consists of viscoelastic and viscoplastic components in series (Figure 5), in which the deformation gradient is decomposed into the viscoelastic, $F^{VE}$ , and viscoplastic, $F^{VP}$ , components as Eq. 1:

$$F=F^{VE}F^{VP}$$ (1)

The viscoelastic component was modeled using a Maxwell–Wiechert model with viscosity parameters of $\eta_{1}$ and $\eta_{2}$ to capture two separate low and high-strain rate viscoelastic mechanisms within the microstructure of the bone. The rate sensitivity at lower and higher strain rates is likely due to the collagen fibers’ nature and the presence of interstitial fluid within the microstructure, respectively (Johnson et al., 2010). The total Cauchy stress, T , of the model, can be calculated by simply summing the individual Cauchy stress as, Eq. 2:

$$T=T^{E0}+T^{E1}+T^{E2}$$ (2)

where the Cauchy stress of the fully elastic branch, $T^{E0}$ , is calculated as, Eq. 3:

$$T^{E0}=\frac{F^{VE}S^{E0}{F^{VE}}^{T}}{\det\left(F^{VE}\right)}$$ (3)

and the Cauchy stress of the viscoelastic branches, $T^{E1}$ and $T^{E2}$ , can be obtained by employing the central difference method to the deviatoric viscoelastic strain rate, ${\dot{\varepsilon }}^{{VE}^{\prime }}$ , expressed as, Eq. 4

$${\dot{\varepsilon }}^{{VE}^{\prime }}=\frac{3}{2\eta_{i}{\left(\frac{BV}{TV}\right)}^{k}}T^{{{VE}^{\prime }}_{i}}+\frac{3}{2\mu_{0}^{i}{\left(\frac{BV}{TV}\right)}^{k}}{\dot{T}}^{{VE}_{i}}\text{ For }i=1,2$$ (4)

where the bone is assumed to be an isotropic material with the shear, $\mu_{0}^{i}$ , and bulk, $k_{0}^{i}$ , moduli, Eqs 5, 6:

$$\mu_{0}^{i}=\frac{E_{0}^{i}}{2\left(1+\nu \right)}$$ (5)

$$k_{0}^{i}=\frac{E_{0}^{i}}{3\left(1-2\nu \right)}$$ (6)

In this way, the second Piola–Kirchoff stress of the fully elastic branch, $S^{EO}$ , in Eq. 3 can be calculated as, Eq. 7

$$S^{EO}=2\mu_{0}^{0}{\left(\frac{BV}{TV}\right)}^{k}\varepsilon^{{VE}^{\prime }}+K_{0}^{0}tr\left(\varepsilon^{VE}\right)I$$ (7)

where I is the identity matrix. Also, the deviatoric viscoelastic strain, $\varepsilon^{{VE}^{\prime }}$ , can be obtained as, Eq. 8

$$\varepsilon^{{VE}^{\prime }}=\varepsilon^{VE}-\frac{1}{3}\;tr\left(\varepsilon^{VE}\right)I$$ (8)

Similarly, after calculating the total Cauchy stress from Eq. 2, the deviatoric Cauchy stress, $T^{\prime }$ , can be calculated as, Eq. 9:

$$T^{\prime }=T-\frac{1}{3}\;tr\left(T\right)I$$ (9)

From there, the magnitude, *τ*, and the direction, N , of the deviatoric Cauchy stress can be obtained as, Eqs 10, 11:

$$\tau =\sqrt{\frac{1}{2}tr\left(T^{\prime^{T}}T^{\prime }\right)}$$ (10)

$$N=\frac{1}{\tau \sqrt{2}}T^{\prime }$$ (11)

The model viscoplastic behavior is calculated based on the co-directionality assumption for the plastic flow and the deviatoric total Cauchy stress, which results in the following rate of plastic stretching, Eq. 12

$$D^{VP}={\left(\frac{\tau \sqrt{3}}{S_{0}}\right)}^{m}N$$ (12)

with *m* as the inverse of the slope of the log-log relationship between the strain rate and plastic yield stress and the natural logarithm of $S_{0}$ as the *y*-intercept of this relationship. From there, the rate of change of the plastic deformation gradient is calculated as follows, Eq. 13:

$${\dot{F}}^{VP}=F^{{VE}^{-1}}D^{VP}F$$ (13)

#### 2.2.2 Damage model

The damage model is derived based on the work by Dall’Ara et al. (2013) based on the elastic-plastic-damage model proposed by Garcia et al. (2009), in which a Halfspacewise Hill criterion is defined as, Eq. 14:

$$Y\left(S,D\right)=\sqrt{S:F_{\pm }\;S}-r\left(D\right)$$ (14)

The damage hardening law, which adjusts the yield surface, is defined as, Eq. 15:

$$r\left(D\right)≔1-\left(1-\alpha \right)e^{-kD}$$ (15)

With α: the ratio between the yield and ultimate stress. The fourth-order tensors, $F_{\pm }$ , defines the different yield properties for compression and tension and is expressed as, Eq. 16:

$$F_{\pm }=\frac{1}{TF^{2}}\left(\sum_{i=1}^{3}\frac{1}{{\left(\sigma_{0}^{\pm }\right)}^{2}}M_{i}⊗M_{i}-\sum_{i,j=1;i\#j}^{3}\frac{\chi_{0}^{\pm }}{{\left(\sigma_{0}^{\pm }\right)}^{2}}M_{i}⊗M_{j}+\sum_{i,j=1;i\#j}^{3}\frac{\chi_{0}^{\pm }}{2\tau_{0}^{2}}M_{i}\underline{\bar{⊗}}M_{j}\right)$$ (16)

where $\sigma_{0}^{\pm }$ is the uniaxial tensile and compressive strengths, $\tau_{0}$ is the shear strength, $\chi_{0}^{\pm }$ is the stress interaction coefficients, and *TF* is the tissue function used to scale the model for dense bone (BV/TV > 0.5), Eq. 17:

$$\begin{array}{l} \text{TF}\left(\text{BV}/\text{TV},E_{\max},E_{0},k\right)\\ =\left\{\begin{array}{ll} {\left(\text{BV}/\text{TV}\right)}^{k} & \text{if BV}/\text{TV}\leq 0.5\\ {\left(\text{BV}/\text{TV}\right)}^{k}+\frac{E_{\max}-E_{0}}{E_{0}}{\left(\frac{\left(BV/TV\right)-0.5}{0.5}\right)}^{2k} & \text{if BV}/\text{TV}>0.5 \end{array}\right. \end{array}$$ (17)

***M*** is the second-order fabric anisotropy tensors defined as:

$$M_{i}≔m_{i}m_{i}⊗m_{i}$$ (18)

where *i* = 1,2,3. The eigenvectors ***m***_*i*_ correspond to the normal direction of the orthotropic symmetric planes, and the eigenvalues *m*_*i*_ express the extent of anisotropy since we model the bone as isotropic, *m*_*i*_ = 1.

### 2.3 Homogenized finite element (hFE) model

Based on Pahr and Reisinger (2020), the 24.5 μm μCT data was resampled to a resolution of 0.3185 mm isotropic per voxel (Medtool ver. 1.4™, Dr. Pahr Ingenieurs e.U). We selected this image resolution to represent the in-plane clinical CT data resolution (0.3185 mm) obtained under our ongoing patient study with a slice thickness used in clinical CT images (0.625 mm). For each image voxel, bone mineral density (BMD) was assigned based on an empirical conversion law relating CT HU to a BMD value followed by a threshold operation (390 mg/cm^3^). We used Medtool 1.4™, a computational geometry and 3D mesh generation software library (https://www.cgal.org), to mesh the masked volumes with a tetrahedral mesh (element size = 1.0 mm). Mesh quality was improved using the HealMesh software library (Mauch et al., 2006) geometrical and topological mesh optimization algorithms. Subsequently, each mesh element was assigned a local BV/TV by interpolating the segmented and masked image (Medtool 1.4™).

#### 2.3.1 Material model parameter calibration

The boundary value problem was initially solved for a selected hFE model with a set of initial reference material model parameters. An iterative procedure was employed where the most relevant material parameters were incrementally adjusted from their reference values in the direction in which they would reduce the RMSE of the simulated load-displacement curve relative to the experimentally measured (Burke et al., 2018). The process was stopped when RMSE was equal to or lower than 5%. The initial trial values of the viscoelastic, viscoplastic cortical bone and damage model parameters were taken directly from Siegel et al. (2012). To reduce the degrees of freedom in the calibration process, we chose to independently calibrate only material model parameters deemed relevant for applying quasi-static, monotonic compressive loading. The following set of material model parameters were adjusted proportionally. 1) The Young’s moduli for the viscoelastic branches, $E_{0}^{1}$ and $E_{0}^{2}$ , were adjusted by the same factor as that of the equilibrium response, $E_{0}^{0}$ . The viscosity parameters for the viscoelastic branches, η_0_^1,^ and η_0_^2^, were also adjusted by the same factor as *E*_0_^0^ with an additional factor of 1/100 to reduce rate-dependency effects further for our quasi-static study. Similarly, the uniaxial tensile strength σ_0_^+^ and shear strength τ_0_ were reduced by the same factor as the uniaxial compressive strength σ_0_^−^. Non-dimensional parameters such as the parameter governing the relationship between strain rate and yield stress *m*, the stress interaction coefficients χ_0_^∓^, the two parameters defining the viscous damage behavior χ_0_^∓^ and ζ, and the ratio between the yield and ultimate stress α, were kept constant. The resulting material model parameters for each vertebra are reported in Table 2.

**TABLE 2:** Optimization-based model parameters computed for the viscoelastic, viscoplastic, and damage models.

Material constants for cortical bone viscoelastic and viscoplastic model parameters						
Elasticity					Plasticity	
$E_{0}^{0}\left[GPa\right]$	$E_{0}^{1}\left[GPa\right]$	$E_{0}^{2}\left[GPa\right]$	$\eta_{0}^{1}\left[MPa∙s\right]$	$\eta_{0}^{2}\left[kPa∙s\right]$	$S_{0}\left[MPa\right]$	m
2.9	0.78	4.18	0.24	0.40	140	18.24

Material constants for damage model								
$\sigma_{0}^{-}\left[MPa\right]$	$\sigma_{0}^{+}\left[MPa\right]$	$\tau_{0}\left[mPa\right]$	$\chi_{0}^{-}$	$\chi_{0}^{+}$	$m_{\upsilon }$	α	$E_{\max}\left[GPa\right]$	k
30.9	21.1	17.1	0.333	−0.246	4	0.6667	2.9	1.5

#### 2.3.2 Finite element (FE) model simulations

A C++ driver to model the quasi-static loading of vertebrae under compression was implemented within ∑MIT (http://summit.mit.edu), a computational solid mechanics framework developed by Professor Raúl Radovitzky’s group (Radovitzky et al., 2011), providing parallel computing large-scale simulations. For the simulation, we imposed a monotonically increasing uniaxial compressive displacement along the craniocaudal direction on the upper surface of the vertebral mesh model and an encastre boundary condition on the inferior surface of the vertebral mesh model. At each iteration step, the imposed displacement and compressive force resultant, calculated as the sum of the residuals at each node of the cranial boundary, were saved, resulting in a simulated compressive load-displacement curve. The apparent vertebral stiffness (K) was calculated as the slope of the curve linear region. Failure strength (F_max_) was computed as the maximal compressive load predicted. In addition, the spatial distribution and magnitude of local stresses, strains, and simulated damage magnitude were computed. The simulation was set to stop when the local damage *D* of any quadrature point reached 1 (completely damaged). The model damage simulations at failure corresponding to the nine vertebrae for which load-displacement curves were illustrated in Figure 3 are presented in Supplementary Figure SA1.

#### 2.3.3 Mesh resolution study

The boundary value problem was solved initially for the hFE 1 mm mesh model, a mesh size selected based on our previous studies (Stadelmann et al., 2020). To study the effect of mesh size on the results, we produced additional FE mesh models at 0.5 mm and 2 mm for each vertebral sample. The hFE model was employed for each vertebral sample to simulate vertebral strength and stiffness at each mesh size. Figure 3 shows the load-displacement curves of 10 sample cadaver vertebral bodies with 2, 1, and 0.5 mm mesh sizes. Quantitative values of vertebral strength and stiffness at each mesh size are detailed in Supplementary Table SA1.

### 2.4 Vertebral specific material model parameter calibration

Similar to the previous section, we initially solved the boundary value problem for the hFE model 1 mm mesh model with the initial reference viscoelastic, viscoplastic cortical bone, and damage model based on material model parameters for bone (Johnson et al., 2010; Dall’Ara et al., 2013). We used an iterative procedure where the most relevant material parameters were incrementally adjusted from their reference values in the direction in which they would reduce the RMSE of the simulated load-displacement curve relative to the experimentally measured one (Stadelmann et al., 2020) to less than 5%. For complete details, see Supplementary Appendix. The resulting material model parameters are reported in Table 2.

### 2.5 Optimization method for computing “global” material parameters

The vertebra’s geometry and internal microstructure affect its ability to withstand loads, specifically its stiffness and strength values. Each of the study’s ten vertebrae varies in geometry and internal microstructure parameters due to bone metastasis and individual variation in geometry and material properties. We aimed to investigate whether a single set of material parameters could be applied to simulate vertebral strength and stiffness across the three bone metastasis types. Such a model is highly desirable as it will obviate the need to achieve patient-specific calibration. To achieve this goal, we define an objective function as:

$$Obj=\lambda \times RMSE+\left(1-\lambda \right)\times SD$$ (19)

where RMSE (Root Mean Squared Error) represents the average error across all samples, and SD denotes the standard deviation as a measure of the variance or spread of errors across all the samples. *λ* is a hyperparameter that controls the trade-off between the two objectives, here set as = 0.5. The normalized error between predicted and experimental mechanical responses for each vertebra is defined below and obtained using the trapezoidal rule, which approximates the area under the curve by dividing it into trapezoids and summing their areas, Eq. 19:

$$e_{i}=\int_{0}^{\delta_{failure}^{i}}\delta {\hat{F}}^{i}d\delta$$ (20)

where ${\delta \hat{F}}^{i}=\left(F_{hFE}^{i}-F_{\exp}^{i}\right)/F_{\exp}^{i}$ is the normalized error, and $\delta_{failure}^{i}$ is the displacement at the failure point for the *i*th vertebra. Normalizing the error (dividing the error for each vertebra by the area under the experimental force-displacement curve of that vertebra) ensures consistency in comparing errors across different experimental data sets. This process is particularly important as we process heterogeneous data sources with different stiffness and strength value ranges. The material model parameters are selected within the material parameters obtained for each vertebra. This strategy ensures that the initial parameter values are within a feasible range based on the available data while allowing for exploration of the parameter space to find a common set of parameters applicable to all vertebrae.

We performed the FE analysis for all ten vertebrae using the selected material model parameters, calculated the error between the FE predictions and experimental measurements using Eq. 20, and evaluated the selected set of material model parameters performance by calculating the objective function Eq. 19. We used the pair of material parameters and the calculated objective function to train a surrogate model, with the surrogate model implemented using a three-layer neural network framework provided by PyTorch having two hidden layers containing 20 neurons and 10 neurons, respectively. Using the trained surrogate model computed, we applied the gradient descent method to optimize the material parameters to minimize the objective function. Using the trained surrogate model for an initial guess, we computed the gradient of the objective function concerning the material parameters and adjusted the parameters iteratively in the direction that minimizes the objective function. This process was performed using PyTorch’s automatic differentiation capabilities, efficiently computing gradients with respect to the material parameters instead of the trained surrogate model’s internal parameters (weights and biases). Table 2 presents the obtained values.

### 2.6 Statistical analysis

Statistical analysis was performed in JMP Pro (14.3, SAS Institute, Inc.). Descriptive statistics were used to summarize the specimens’ demographic, bone, and mechanical properties. The strength and stiffness data were not normally distributed based on the normality test results (Shapiro-Wilk test). Based on the test for unequal variability between the mesh size data, Welch’s ANOVA was used to test for the effect of 1) bone metastases on the experimental strength and stiffness of the osteolytic and osteosclerotic vertebrae, 2) mesh size on the differences in strength and stiffness and 3) computed errors with respect to the experimental data. Post hoc comparisons were performed using the non-parametric Steel-Dwass test.

The sampling of multiple vertebrae per spine can introduce clustering (non-independence) of the data. We fitted linear mixed-effects models (LMMs) under different assumptions about the correlation structure among segments from the same spines to test this effect. According to the Akaike information criterion (AIC), the independence structure best fits the data (O’Brien and Fitzmaurice, 2004). We applied linear regression to test the association between the simulated and measured strength and stiffness based on this finding. Statistical significance was set at the 5% level.

## 3 Results

### 3.1 Experimental study

Vertebral demographics, primary cancer, bone material, and vertebral mechanical properties are listed in Table 1. Corresponding experimental load-displacement curves are presented in Figure 2. Osteolytic vertebrae strength, a mean (standard deviation) of 2.65 (1.09) kN, and stiffness 10.75 (1.51) kN/mm were lower than osteosclerotic vertebrae [7.44 (2.43) kN, *p* = 0.0069 and 14.45 (2.96) kN/mm, *p* = 0.0405, respectively]. The vertebrae with mixed bone metastases exhibited high strength, 10.80 kN, and stiffness, 36.84 kN/mm.

### 3.2 Bone metastasis affects vertebral structure

**FIGURE 1:**
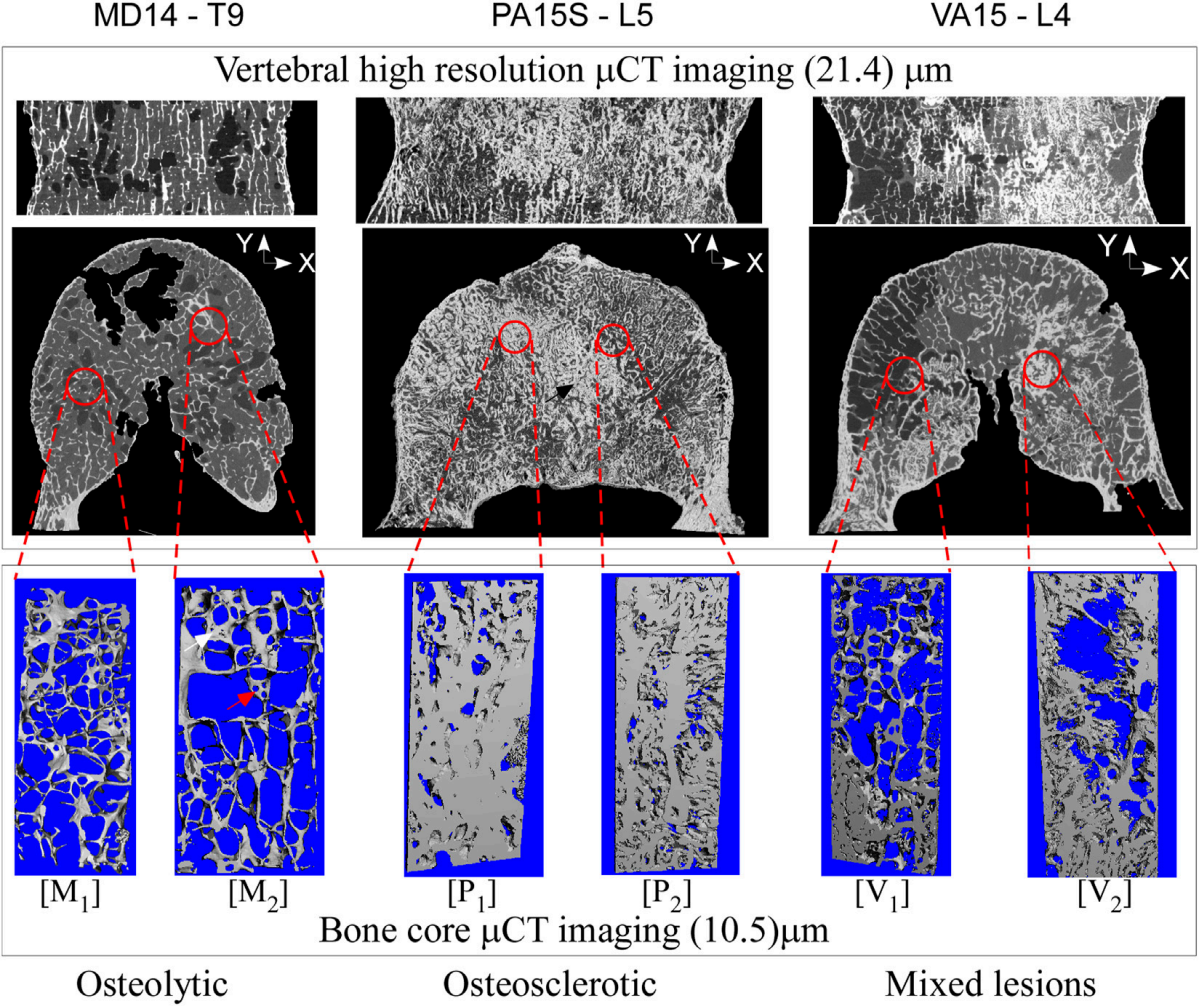
High-resolution CT images of vertebral bodies with osteolytic, osteosclerotic, and mixed bone metastatic illustrate bone metastasis’s remarkable effect on bone architecture. In the osteolytic vertebra, the bone architecture shows loss of bone interconnection with loss of bone interconnection and tissue fenestration (marked by the red arrow (image M_2_) leading to low BV/TV. In the osteosclerotic vertebra, the bone architecture shows a nearly solid bone tissue (M_2_), resulting in high BV/TV, observed in the axial vertebral images as thick bands of dense bone. The mixed lesion vertebra shows areas of high BV/TV (image V_2_), typical of osteosclerotic vertebra, with areas of low bone BV/TV (image V_1_), typical of osteolytic vertebra.

**FIGURE 2:**
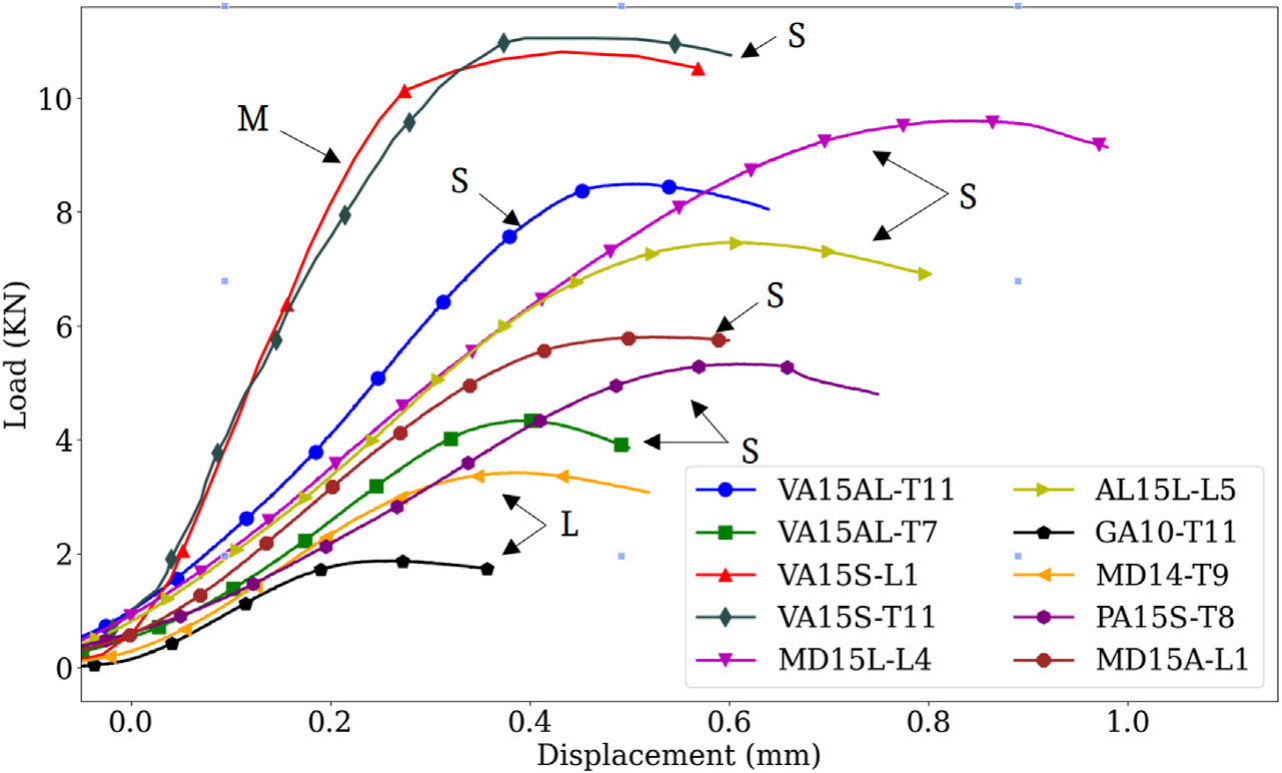
Comparison of experimental load-displacement curves showing bone metastases effect on the vertebral mechanical behavior. Osteosclerotic vertebrae had predominantly high strength and stiffness compared to osteolytic vertebrae. Note, however, the high variation in strength and stiffness between osteosclerotic vertebrae. Although containing regions of osteolytic bone, the high strength and stiffness exhibited by the mixed lesion vertebrae exemplify the clinical difficulty in classifying the fracture risk of this type of bone lesion. S, osteosclerotic, L, osteolytic, M, mixed bone metastases.

Presents sagittal and axial *μ*CT image data for an osteolytic, mixed, and osteosclerotic vertebral sample used in the study. The osteolytic vertebrae presented a thinning of the cortex and rarefaction of the trabeculae (Figure 1: M1 and M2) with focal bone loss (cavities) through the entire Vertebra (Figure 1). Osteosclerotic vertebrae demonstrate a marked increase in BV/TV, as can be observed from the near solid tissue (Figure 1: P2 and P2). Per its classification, Vertebra with a mixed lesion demonstrates regions of high bone density with regions containing lytic lesions (Figure 1: V1 and V2). Descriptive analysis found osteosclerotic vertebrae with higher bone mineral density (BMD), mean (standard deviation) value of 213.19 (60.44) mgHA/cm^3^ and bone volume fraction (BV/TV), 12.80 (7.13)%, than osteolytic vertebrae, (108.095 (23.75) mgHA/cm^3^ and 9.06 (0.70)%, respectively). ANOVA analysis found these differences not statically significant at the 5% level. The mixed lesion vertebrae showed high BMD (324.68) mgHA/cm^3^ and BV/TV (18.61)%, a value significantly higher than osteolytic vertebrae BMD (*p* = 0.039) Figure 2.

### 3.3 Mesh resolution study

**FIGURE 3:**
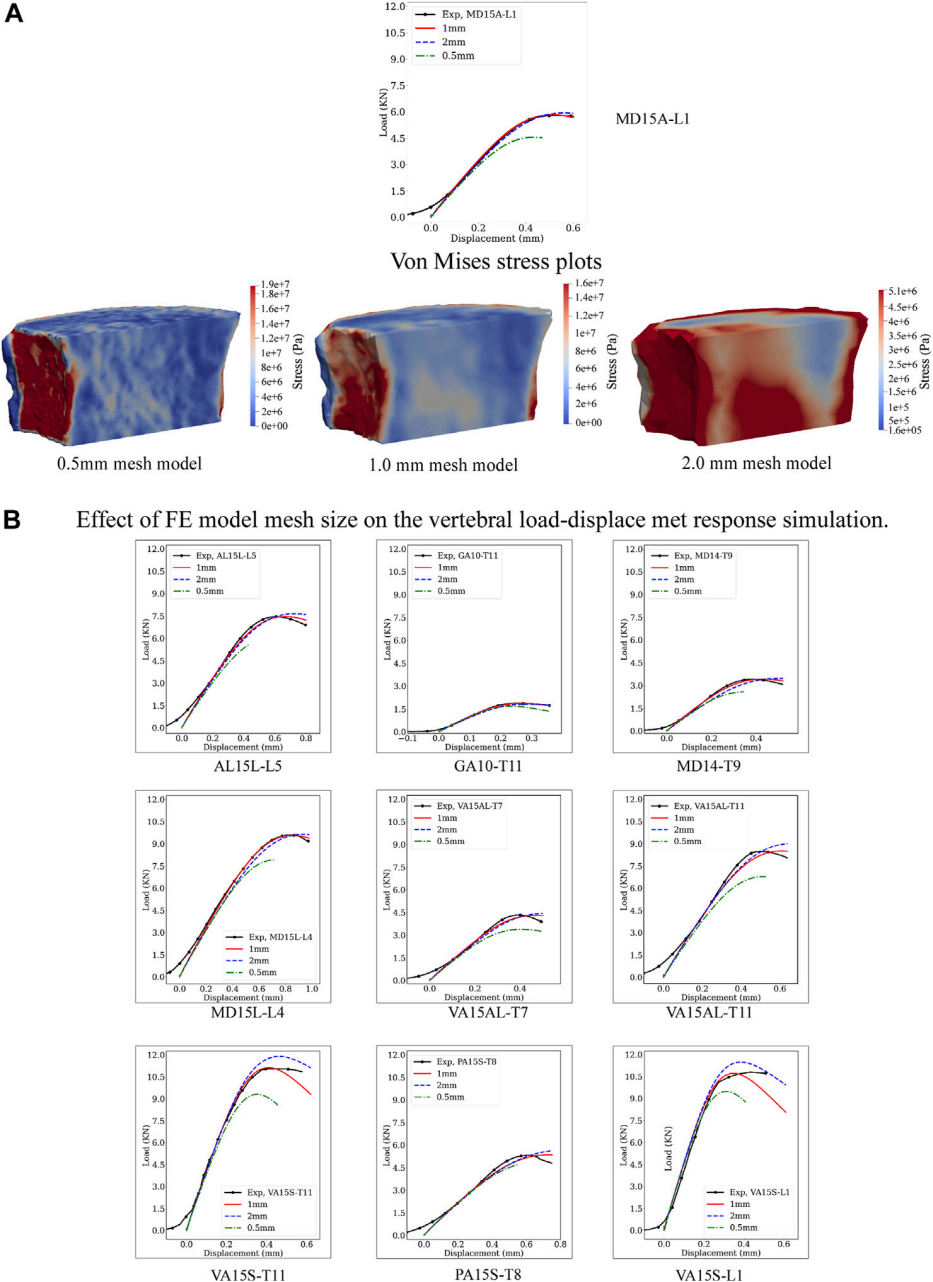
Effect of the computational model mesh size on the model’s prediction of vertebral load-displacement response. **(A)** Compares the load-displacement curves simulated at 0.5, 1, and 2 mm element mesh size with the measured curve for the MD-15A-L1 specimen. For each mesh size, a pictorial illustration presents the Von Mises stress contours with the stresses computed as a criterion for yielding or fracture of ductile materials under complex loading. The finer mesh, 0.5 mm, although providing greater details for the stress patterns in the vertebral volume **(A)**, resulted in the “softening” of the simulated response compared to the experimental curve. By contrast, the coarser mesh, 2 mm, over-predicted the experimental vertebral strength with lower accuracy in simulating stiffness. These differences were consistent for each study vertebrae **(B)**. The optimality of the 1 mm mesh simulation across the simulated specimen highlights the need for careful selection of mesh size based on bone architectural features.

Figure 3 presents experimental and predicted load-displacement curves at 2, 1, and 0.5 mm mesh models for MD15A-L1. Corresponding mid-sagittal von Mises stresses at vertebral strength are presented for each mesh size simulation. Supplementary Tables SA1, SA2 detail the specimen-specific strength, stiffness, and corresponding error (%) values at 2, 1, and 0.5 mm mesh models.

#### 3.3.1 Strength simulations

FE-simulated strength, a mean (standard deviation) of 6.82 (3.16) kN, showed excellent agreement with the measured strength, 6.80 (3.16) kN, Supplementary Table SA1, simulation error compared to the experimental strength, Root Mean Squared Error (RMSE) = 0.04. Mesh refinement, 0.5 mm, underestimated strength, 5.86 (2.81) kN/mm Supplementary Table SA1, with higher simulation error (RMSE = 0.42). A coarser mesh size, 2 mm, overestimated strength, 7.33 (3.48), Supplementary Table SA1, (RMSE = 0.32). ANOVA analysis found these differences significant (*p* < 0.0001). Post-test comparisons showed 0.5 mm and 2 mm RMSE values significantly higher than the 1 mm model (*p* = 0.0005 and *p* = 0.0017, respectively), with 0.5 mm model RMSE higher than the 2 mm model (*p* = 0.0160).

#### 3.3.2 Stiffness simulations

The predicted stiffness at 1 mm mesh model, 18.50 (11.47) kN/mm, closely agreed with the experimental values, 18.60 (11.23) kN/mm, Supplementary Table SA2, with RMSE = 0.23. Either refinement, to 0.5 mm or coarsening, to 2 mm, of the mesh models resulted in higher predicted stiffness [19.48 (11.23), RMSE = 0.75 and 19.86 (11.79), RMSE = 1.21, respectively] kN/mm, Supplementary Table SA2. ANOVA analysis found these differences significant (*p* < 0.0192). Post-test analysis showed the error values for the 2 mm mesh higher than the 1 mm model (*p* = 0.0101).

### 3.4 hFE predicts metastatic vertebrae strength and stiffness

**FIGURE 4:**
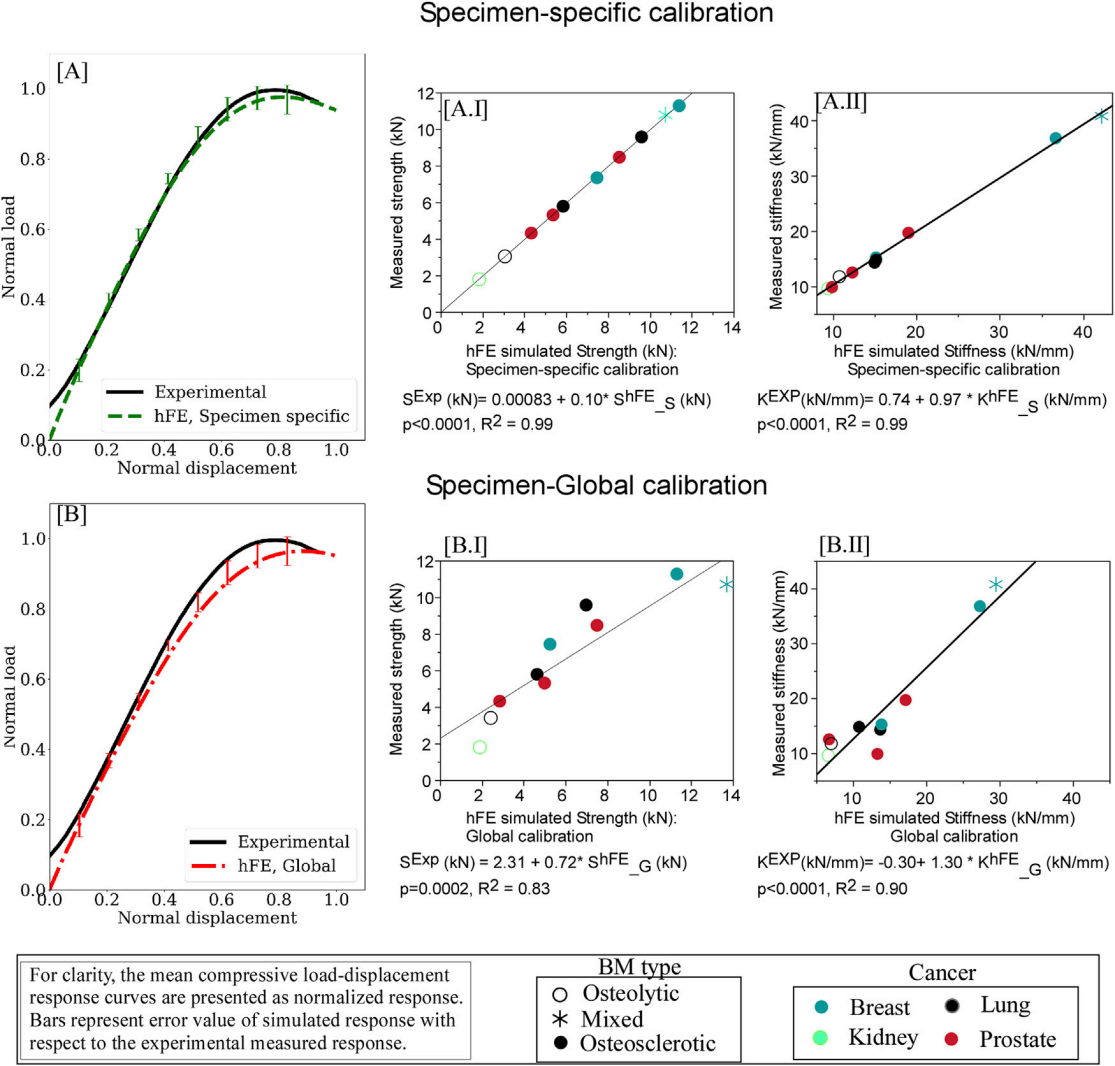
hFE damage-based model strongly predicts pathologic vertebrae experimental load-displacement response using specimen-specific **(A)** and optimization (“global”) **(B)** approaches. For each curve, the data is presented normalized with the peak force and displacement at failure. The error bars presented for the simulated load response represent standard deviation values. Regression analysis found the predicted strength **(AI)** and stiffness **(AII)** values computed for the specimen-specific material model calibrations in excellent agreement with the measured strength and stiffness values. Regression analysis demonstrated that employing “global” material calibration yielded strong agreement between the FE simulation and the measured values for vertebral strength **(BI)** and stiffness **(BII)**.

Figure 4 presents the mean load-displacement curve for experimental and simulated specimen-specific (Figure 4A) and global (Figure 4B) experiments. Detailed values for specimen-specific and global material calibration-based prediction of strength and stiffness values and corresponding computed error concerning experimental values are detailed in Supplementary Tables SA3, SA4, respectively.

#### 3.4.1 Specimen-specific material calibration

**FIGURE 5:**
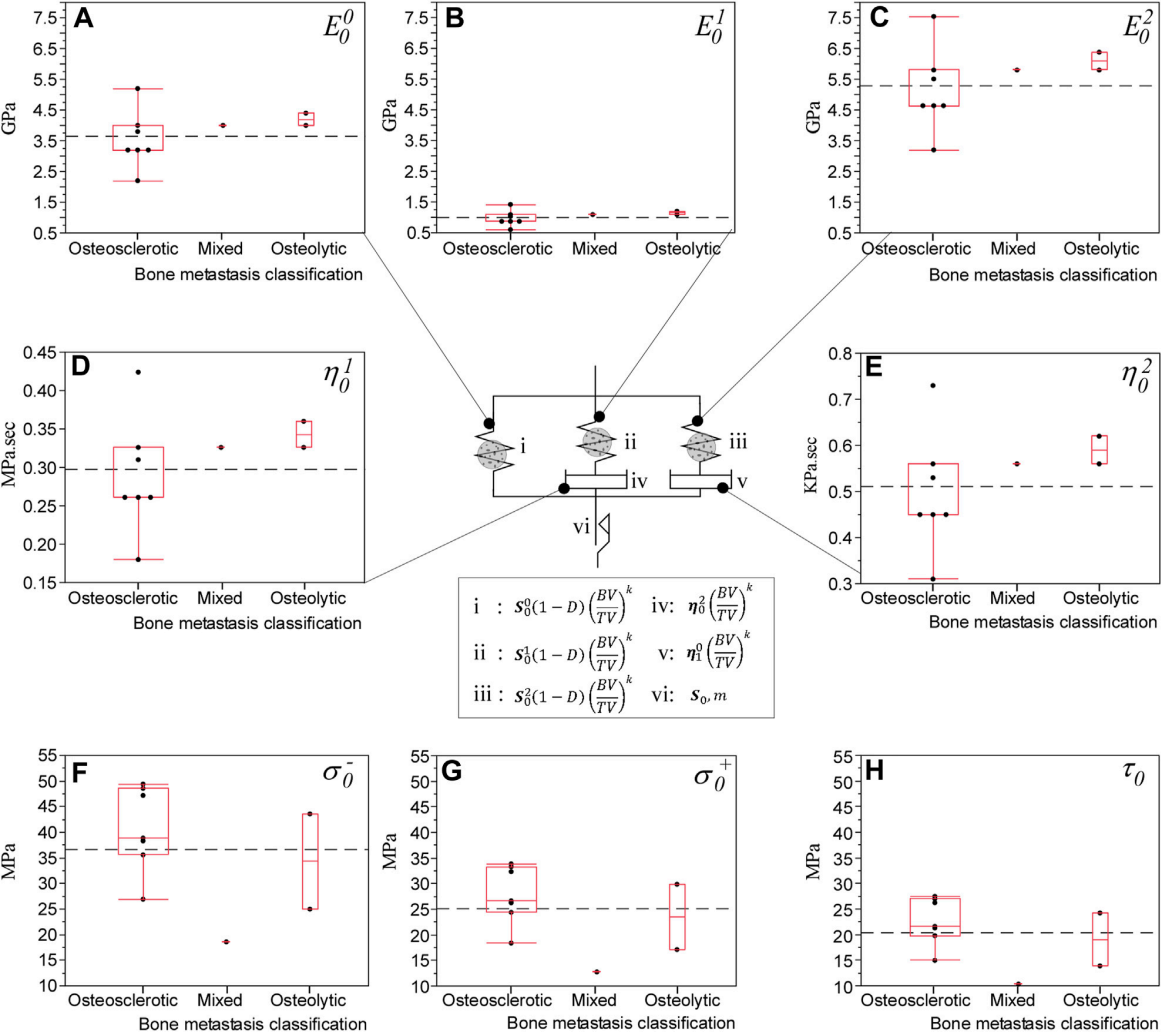
hFE predicted the effect of bone metastasis on the rheological damage-based bone model material parameters that closely match experimental findings (Stadelmann et al., 2020). The rheological damage-based bone model underlying the computational approach consists of a long-term elastic modulus, $E_{0}^{0}$ (Gpa) **(A)**, in parallel with the bone’s low and high rate viscoelastic behavior, modeled via elastic elements ( $E_{0}^{1}$ and $E_{0}^{2}$ ) Gpa, **(B,C)** in series with dashpot elements representing low and high rate viscous strain, $\eta_{0}^{1}$ (MPa∙s) and, $\eta_{0}^{2}$ (kPa∙s), **(D,E)**. The bone’s plastic deformation is modeled via a rate-independent plastic behavior (element vi). The computational model predicted the osteosclerotic bone with lower elastic modulus and time-dependent deformation, which agrees with our previous experimental study (Stadelmann et al., 2020). At failure, the computational model predicted osteosclerotic bone with higher compressive, σ_0_^−^, tensile σ_0_^+,^ and shear stress τ_0_ (sub-plots **F–H**), than the osteolytic bone, reflecting the effect of lower BV/TV in osteolytic bone on the failure of the vertebrae.

Regression analysis showed specimen-specific material calibration simulations explained 99% of the variance of measured strength (*p* < 0.0001, Figure 4A.I) and stiffness (*p* < 0.0001, Figure 4A.II). The rheological model (Figure 5) showed the osteosclerotic vertebrae long-term modulus, $E_{0}^{0}$ , [3.5 (0.92)]GPa, viscoelastic low-, $E_{0}^{1}$ : [0.96 (0.25)]GPa and high-, $E_{0}^{2}$ : [5.19 (1.35)]GPa modulus, and viscoelastic strain rate low-, $\eta_{0}^{1}$ : [2.89 (0.76)MPa∙s, and high, $\eta_{0}^{2}$ : 0.49 (0.13)kPa∙s] parameters lower than osteolytic vertebrae elastic [ $E_{0}^{0}:$ 4.2 (0.28), $E_{0}^{1}$ : 1.15 (0.07), and $E_{0}^{2}$ : 6.09 (0.41)]GPa and viscoelastic [ $\eta_{0}^{1}$ : 3.43 (0.24)MPa∙s and $\eta_{0}^{2}$ :0.59 (0.04)kPa∙s] parameters, respectively, Supplementary Table SA5. Mixed-lesion vertebra model parameters were comparable to osteolytic vertebrae.

#### 3.4.2 Global material calibration

Regression analysis demonstrated that the simulation using global material calibration explained 83% of the variance in measured strength (*p* = 0.0002) and 92% in stiffness (*p* < 0.0001), Figure 4B. Compared to the specimen-specific calibration, analysis of variance (ANOVA) found the “global” model reduction in strength prediction accuracy to be significant (*p* = 0.0060). We found no such statistically significant difference in the stiffness prediction at the 5% level.

### 3.5 FE simulation suggests bone metastasis type affects bone damage evolution differentially

**FIGURE 6:**
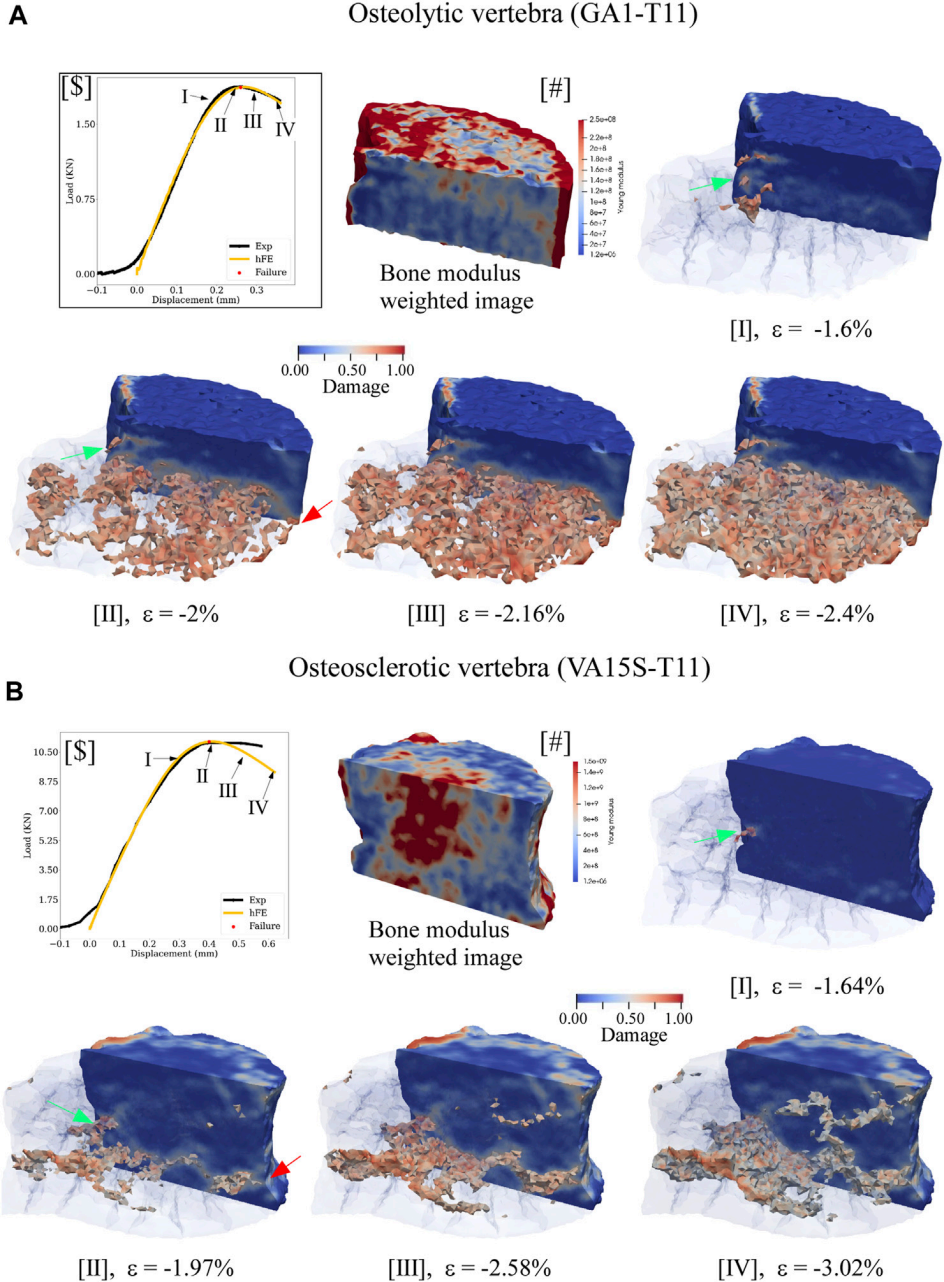
hFE model illustrates bone metastasis effect on the progression of simulated damage for osteolytic **(A)** and osteosclerotic **(B)** vertebrae for pre-failure, failure, and post-failure load states. For ease of visualization, the medial half of the vertebral body was made transparent to view the spatial evolution of damage within the vertebral body. The lateral half was rendered solid with blue, indicating no damage at a specific location. In osteolytic vertebrae **(A)**, the FE simulation indicated bone damage to initiate at the posterior cortex at yield (green arrow, **A**.I) and to accumulate within the region of low modulus (**A**.II) to failure (Figure 5c.II). In the osteosclerotic vertebra, **(B)**, damage initiates at the posterior cortex at yield (**B**.I, green arrow), evolving confined to a narrow region of low bone modulus within the body at failure. Note that this process largely bypasses regions of high bone modulus (**B**.II). For both bone metastasis types, failure was simulated to occur once the simulated damage within the vertebral bone network coalesced with damage at the anterior and posterior vertebral cortex (green and red arrows). Post-failure progression of the simulated damage appears confined to the region of existing damage in the osteolytic vertebra (**A**c.III,IV). In the osteosclerotic vertebra, the model suggests that simulated damage evolves within the pre-existing damaged region (**B**.III) with increasing involvement of regions with a heterogeneous distribution of bone modulus (**B**.III,IV).

In the osteolytic vertebra, the FE simulation suggested the damage initiating at yield strength at the posterior cortex (Figure 6A.I), evolving within the vertebral body through a large region of low bone modulus to failure (Figure 6A.II). In the osteosclerotic vertebra, the FE simulation suggested damage initiated at yield strength at the posterior cortex (Figure 6B.I), the damage evolving largely confined to regions of low bone modulus principally bypassing regions of high bone modulus (Figure 6B.II). Upon reaching simulated failure, the simulated damage was confined to the pre-damaged region in the osteolytic vertebra (Figure 6A.III,IV) while, in the osteosclerotic vertebrae, the simulated damage evolved within bone regions with low and heterogeneous distribution of bone modulus (Figure 6B.III,IV).

## 4 Discussion

This study developed a constitutive, damage-based finite element model to simulate the strength and stiffness of cadaveric pathologic vertebrae containing osteolytic, osteosclerotic, and mixed bone lesions. Derived from CT data comparable to standard clinical CT image resolution and implemented within the ΣMIT computational solid mechanics framework (Radovitzky et al., 2011), the model accurately predicted the vertebrae’s strength and stiffness under applied compressive loading independent of the metastatic bone lesion type. We further demonstrated the feasibility of a machine learning approach to establish a unified set of material calibration parameters, providing a strong prediction of pathologic vertebral strength and stiffness. This finding suggests that such a unified set, once validated in a larger data set, might reduce the need to incorporate density phantoms during patient radiation treatment planning, which would require significant investment in the recertification of CT protocols. The FE model damage simulation suggested that osteolytic and osteosclerotic lesions differentially affect damage evolution within the bone network.

### 4.1 Selection of vertebral specimens for hFE model validation

We aimed to simulate the measured compressive strength and stiffness of human vertebrae with osteolytic, osteosclerotic, and mixed bone metastases from a group of 45 cadaveric vertebrae that were obtained from donors with breast, lung, renal, and prostate cancers and mechanically tested in our previous study (Stadelmann et al., 2020). The vertebrae selected demonstrated either markedly higher strength, stiffness, and BMD values, for example, VA15-S-T11 [(+84.5, +49.8, and 73.1)%, respectively), markedly lower, [GA1-T11: (−69.7, −60.7 and −50.4)% respectively] or asynchronous values for mechanical and material properties, [PA15-T8 (−13.8, −59.4, +30.8)%, respectively], than the median strength and stiffness values measured for the 45 vertebrae tested (Stadelmann et al., 2020). Although little data exists on the strength of human vertebrae with osteosclerotic or mixed bone metastases, the current study osteolytic vertebral strength, a mean (SD) of 2.6 (1.1)kN (Stadelmann et al., 2020), agrees with strength values reported for osteolytic cadaver vertebrae by Costa et al. (2019). Therefore, the pathologic vertebrae selected for this study form a challenging sample for validating the FE framework proposed.

### 4.2 Establishing the hFE modeling approach

We postulated that integrating bone tissue viscoelastic and viscoelastic response may improve the computational damage model’s ability to simulate the bone metastases-mediated changes in vertebral strength and stiffness. For this study, we modified the elastic element in each of the viscoelastic model branches to incorporate 1) the damage evolution model (Dall’Ara et al., 2013) to capture the spatial bone structure material softening (Section 4.2) and 2) the nonlinear relationships between trabecular bone material properties (Section 4.1) introduced via bone fraction value (BV/TV) (Zysset, 2003) within the model’s elasticity-density relationship for trabecular bone tissue.

Based on vertebral-specific material calibration, the resulting computational framework successfully explained 99% of the variation in the vertebrae measured strength and stiffness invariant to the type of bone metastases. This prediction improves upon our previous hFE model (Stadelmann et al., 2020) employing “state of the art” FE modeling of human vertebrae mechanical behavior (Pahr et al., 2014), which explained 71% of the variance in the current specimens’ measured strength (*p* = 0.0023) and 52% of measured stiffness (*p* = 0.0180). This confirms that the FE model, as implemented, captures the factors that determine strength and stiffness.

#### 4.2.1 Mesh size affects predicted vertebral strength and stiffness

In agreement with Jones and Wilcox (2007), mesh size affected our model strength and stiffness simulation accuracy. Regression analysis showed the 1 mm calibrated mesh size to yield an excellent agreement between the predicted strength, stiffness, and measured values. Higher mesh size, 2 mm, affected higher averaging of the bone microstructure and thickening of the vertebral cortex region, likely to yield higher bone density per element. This increase resulted in the model’s overestimated simulation strength, a mean of 4.1%, and degraded accuracy (Supplementary Table SA1). Refining mesh size to 0.5 mm yields higher structural fidelity for bone microstructure and vertebral cortex anatomical detail. Although the finer mesh model is expected to produce improved prediction, the higher mesh model underestimated measured strength, a mean of −17.8%, with significantly lower simulation accuracy, Supplementary Table SA1. We assume that the finer mesh contains stiffer and softer elements due to the broader range of local bone volume densities leading to “localized” damage accumulation within the finer mesh regions of low density. This “localized” damage affects higher numerical instability within the mesh, which “softens” the simulated stress-strain response, a finding in agreement with Werner et al. (2019) reporting FE mesh refinement to soften the post-yield behavior of trabecular bone under large deformation. Given these findings, we elected to perform our simulations at 1 mm mesh element size.

#### 4.2.2 Optimization approach for establishing FE model parameters

Capturing the spatial variation in the bone tissue density-elasticity relationships requires mapping CT Hounsfield unit (HU) data to bone density values using a calibrated bone density phantom. However, introducing the density phantom in the imaging field will require re-certifying the radiotherapy planning and imaging protocols for each CT imaging and clinical linear accelerator system, a highly costly and complicated process. Phantomless calibration is based on skeletal muscle or adipose tissues’ known linear attenuation properties to create a standard calibration curve for converting trabecular HU to vBMD the HU values (Gudmundsdottir et al., 1993). Although this technique strongly correlates with phantom-based vBMD analysis in non-cancer patients, it has not been validated in cancer patients, and its utility has yet to be shown to be generalized on multiple CT scanners. Asynchronous calibrations in which the bone-density phantom-based calibration is performed daily as part of the CT system quality assurance separate from the patient scan may alleviate some of these challenges. We, therefore, investigated whether establishing a single set of “optimized” material parameters based on initial asynchronous calibrations using a bone density phantom could simulate vertebral strength and stiffness across the wide range of bone metastases tested in this study.

For this purpose, we applied a “Surrogate optimization” approach (Espinosa et al., 2023) to find a single set of parameters to minimize the summation of errors across all samples while ensuring that the error for each sample is within a similar range. This resulting common objective creates an approximation model that mimics the behavior of the true objective function when the objective function is computationally expensive, time-consuming, or difficult to evaluate directly, allowing for faster parameter space exploration. For this purpose, we used neural networks to construct the surrogate model with the optimization algorithm leveraging gradient-based optimization techniques to find a single set of material model parameters that minimizes error for all ten vertebrae while ensuring that the error is not getting too large. The optimized “global” model explained 83% of the measured strength variance (*p* = 0.0002) and 92% of the measured stiffness variance (*p* < 0.0001).

A possible explanation for the model’s stronger prediction for stiffness vs. strength might be associated with the power coefficient *k* (Eq. 4) assumed to be the same for the model’s morphology-elasticity and morphology-yield relationships. Although stiffness, yield, and ultimate strength of trabecular bone were reported to be strongly correlated (Goulet et al., 1994), it may be necessary to define *k* differently for the two relationships. Overall, this work supports the promise of the global parameter approach to simulate vertebral body mechanics at an actionable level of confidence.

### 4.3 Bone metastases phenotype affects the pattern of vertebral bone damage

The effect of bone metastases type on bone damage accumulation underlying the failure and post-failure behavior of pathologic vertebrae is poorly understood. Atkins et al. (2019) reported that bone metastasis type (osteolytic vs. mixed) affects the pattern of bone tissue microdamage, with pathologic bone exhibiting a significantly higher magnitude of diffuse microdamage compared to the healthy bone for a comparable compressive load. Micro-FE simulations (Choudhari et al., 2016; Atkins et al., 2019) found that regions of higher stress and strain within the pathologic bone correlated with the extent and pattern of diffused micro-damage, the damaged bone tissue showing significantly higher stress magnitude than healthy bone tissue for comparable compressive loading (Atkins et al., 2019). Our FE simulation demonstrates that bone metastases type produces a distinct difference in damage evolution within the vertebral body and that, for both bone metastases types, the damage evolved localized to regions of low bone modulus (Figure 6). Our results are in qualitative agreement with these studies.

The FE framework rheological model revealed osteolytic bone with higher elastic and viscoelastic modulus values than osteosclerotic bone (Figure 5), which agrees with our previous experimental study (Stadelmann et al., 2020). Burke et al. (2016, 2017) reported osteolytic bone metastases alter the bone’s collagen fibril organization and degrade tissue mineralization, likely to increase collagen fibrillar sliding at the collagen/mineral interface under applied stress, affecting the bone plasticity resulting in higher bone microdamage (Vashishth et al., 2000). Our simulations suggest that in osteolytic bone, damage accumulation is primarily associated with inelastic strain accumulation and viscous property, forming new internal surfaces and voids in the tissue (Arthur Moore and Gibson, 2002), affecting the magnitude of diffused damage in the bone.

In osteosclerotic bone, the FE framework rheological model predicted the bone tissue with lower elastic and viscoelastic modulus despite the predicted higher compressive and shear strength at failure. Although little is known about the mechanism of osteosclerotic bone failure, higher bone mineralization, bone connectivity, and thicker bone trabecula yield higher bone volume to tissue volume (Bailey et al., 2022), resulting in a plate-like, dense bone network, which may explain the rheological model prediction of higher strength at the tissue level. The simulation suggested that osteosclerotic vertebrae’s stiffness and overall failure strength were dominated by damage evolution localized to regions of low-bone modulus within the bone network, largely bypassing regions of high modulus associated with high BV/TV. This finding supports our experimental study (Alkalay et al., 2021b), which shows that osteosclerotic and mixed (comprising osteosclerotic and osteolytic regions) vertebrae exhibit similar measured stiffness values. Once failed, the simulation suggested that for both bone metastases types, evolution of bone damage appears localized to the low bone modulus regions within the vertebral volume (Figure 6). Additional studies are required to understand the impact of pathologic bone tissue structure and quality on the damage accumulation process and, ultimately, the failure of pathologic vertebrae.

### 4.4 Study limitations

This study has several limitations beyond the limitations of cadaveric spines.

#### 4.4.1 Experimental protocol

Although our sample size of 10 human vertebrae is small, the vertebral samples were obtained from donors with known spine metastases containing a wide range of clinically observed bone metastases. Our study (Stadelmann et al., 2020) used isolated vertebral bodies with the endplate and posterior elements removed to permit imaging in a small-bore high-resolution CT device and provide a standardized specimen geometry. Computational analysis (Maquer et al., 2014) found that removing the vertebral endplates had a low impact on the vertebrae’s predicted strength and overall damage distribution. However, the degenerative state of the intervertebral disc (Palanca et al., 2023) and the mechanical interaction of the posterior elements (Alkalay et al., 2021) affect the strength, stiffness, mechanical instability and failure patterns (Alkalay et al., 2021a) of pathologic spines. Our current ongoing work aims to incorporate a newly designed computer control mechanical test system designed to allow time-lapse imaging under controlled loading conditions within the XtremeCT II (SCANCO Medical AG, Switzerland) to provide detailed measurements of the effects of bone metastasis on the spine deformation and failure.

Our study used a quasi-static monotonic mechanical loading protocol (Stadelmann et al., 2020). However, monotonic loading has limited fidelity concerning physiological loading conditions. Importantly, the interaction of these loads with the poroelastic intervertebral disc joints (Newell et al., 2020) and, through this interaction, the deformation of the vertebral endplates, will modulate and alter the stress/strain within the affected vertebrae (Alkalay and Harrigan, 2016; Palanca et al., 2023), thereby affecting vertebral failure (Jackman et al., 2016). Recapitulating dynamic testing conditions *in vitro* remains a significant challenge (Costi et al., 2021). Hence, our study offers a narrow simulation of these effects.

As discussed in our previous study (Stadelmann et al., 2020), we could not produce perfectly parallel sections during sample preparation. As a result, we could not produce “zero stress” conditions at the initiation of the test affecting the measurements of vertebral stiffness. Although this does not affect the resulting experimental strength or the model simulation of strength, our study reported experimental and simulated values for vertebral stiffness represent “apparent” vertebral stiffness (Burghardt et al., 2007).

#### 4.4.2 Modeling approach

Although the FE damage-based simulation strongly predicted the experimental measures of vertebral stiffness and strength, we did not directly validate the model’s damage predictions. We sought to predict vertebral strength and stiffness using our damage model as a modeling approach. The accuracy of the prediction validates the approach without explicit measurement of damage, which was not necessary for our study. Previous studies employed cyclic loading experiments to evaluate bone damage (Zysset and Curnier, 1996), yielding loading boundary conditions significantly different from our simulations. We are evaluating controlled loading experiments within high-resolution CT, which, combined with image-based volumetric registration, allow computation of the pattern of internal bone strains to validate the FE damage predictions.

Our FE model employed an isotropic bone volume density parameter to compute the morphology-elasticity relationship. Although bone volume density accounts for 84%–87% of the variation in bone stiffness and strength (Maquer et al., 2015), fabric anisotropy was found to explain a small but appreciable (∼10%) amount of the variation in the elastic properties of bone (Maquer et al., 2015). Given the variation in the location, geometry, and character of metastases, including anisotropy in our constitutive model may further improve the predictability of our “optimization-based” models.

### 4.5 Clinical implications

Vertebral fractures can be devastating events for patients with spinal metastases. Once spinal metastases are identified, there are potential prophylactic therapies that may reduce PVF risk. All are invasive, and treating physicians seek to avoid them unless necessary. Attempts to generate individualized fracture risk predictions have had limited success thus far (Fourney et al., 2011). Although our sample set was small, it includes vertebrae impacted by all metastatic lesion types demonstrating a wide range of vertebral stiffnesses and strengths, permitting characterization of the load at which they fail and, critically, localizing the vertebral region of initial failure. This information, representing an unmet clinical need, may guide localized interventions such as vertebral augmentation to the portions of the vertebral body at greatest risk upon further development and validation. In many patients, the affected vertebral bodies will have lost height due to failure that may be subclinical at presentation. In these cases, methods developed for predicting the fracture risk of intact bodies cannot be expected to succeed. Our approach holds promise for estimating residual strength after the initial fracture. This question of residual strength applies to both pathologic and osteoporotic fractures.

## Figures and Tables

**Table:** 

$\left(\frac{BV}{TV}\right)$	Bone volume fraction, i.e., the volume of mineralized bone per total volume of the sample
**k**	Density-elasticity power law coefficient
$E_{0}^{0}$	Elastic modulus of the long-term equilibrium for a poreless cortical bone
$E_{0}^{1}$	Elastic modulus of the low strain rate viscoelastic for a poreless cortical bone
$E_{0}^{2}$	Elastic modulus of the high strain rate viscoelastic for a poreless cortical bone
$F^{VE}$	Viscoelastic
$F^{VP}$	Viscoplastic
$\eta_{0}^{1}$	Viscosity parameter of the low strain rate viscoelastic for a poreless cortical bone
$\eta_{0}^{2}$	Viscosity parameter of the high strain rate viscoelastic for a poreless cortical bone
**S**_**0**_	Plastic yield stress for a poreless cortical bone
***m***	Inverse of the slope of the trend line between the strain rate and plastic yield stress
$\sigma_{0}^{-}$	Compressive stress
$\sigma_{0}^{+}$	Tensile stress
$\tau_{0}$	Shear stress
**D**	Damage variable
**ε** ^**VE**^	Viscoelastic strain
**ε** ^**VP**^	Viscoplastic strain
${\dot{\varepsilon }}^{{VE}^{\prime }}$	Deviatoric viscoelastic strain rate
$\mu_{0}^{i}$	Shear moduli
$k_{0}^{i}$	Bulk moduli
**I**	Identity matrix
**S**	Stiffness tensor
$S^{EO}$	Second Piola–Kirchoff stress of the fully elastic branch
T	Total Cauchy stress

## Data Availability

The raw data supporting the conclusion of this article will be made available by the authors, without undue reservation.

## Supplementary material

SUPPLEMENTARY FIGURE SA1 hFE model simulated damage accumulation pattern at failure for the study’s remaining osteosclerotic, osteolytic, and mixed lesion vertebrae. For ease of visualization, the medial half of the vertebral body was made transparent to view the spatial evolution of damage within the vertebral body. The lateral half was rendered solid with blue, indicating no damage at a specific location. The marked differences in the damage pattern of the osteosclerotic vertebrae reflect the high variation observed for their load-displacement response. By comparison, the osteolytic vertebra is simulated to fail at a lower value of damage accumulation. The mixed lesion vertebra was simulated to fail at a high level of damage, which may contribute to its measured high strength and stiffness.
