# Supplementary material for: CT-based finite element simulating spatial bone damage accumulation predicts metastatic human vertebrae strength and stiffness
Source: Front Bioeng Biotechnol. 2024 Jul 23;12:1424553. doi: 10.3389/fbioe.2024.1424553 (PMC11300227; doi:10.3389/fbioe.2024.1424553)
Supplement: Supplementary file 1 [file Table1.DOCX]

**Table A.1** Comparison of the measured strength values with simulated values predicted at 0.5, 1, and 2 mm mesh element size. Error values were computed with reference to the experimental data.

|  |  | **Measured vs. predicted strengths** | | | | | | |
| --- | --- | --- | --- | --- | --- | --- | --- | --- |
|  |  | Exp. | 0.5mm mesh  calibration | | 1mm mesh  calibration | | 2mm mesh  calibration | |
| **Spine ID** | BM | S^Exp^  (kN) | S^hFE^_0.5mm_  (kN) | Error__0.5mm_  (%) | S^hFE^_1mm_  (kN) | Error__1mm_  (%) | S^hFE^_2mm_  (kN) | Error__2mm_  (%) |
| **VA15AL-T11** | S | 8.49 | 7.2 | -15.19 | 8.52 | 0.35 | 9.53 | 11.85 |
| **MD15L-L4** | S | 9.59 | 8.35 | -12.93 | 9.58 | -0.10 | 9.99 | 4.28 |
| **VA15AL-T7** | S | 4.34 | 3.29 | -24.19 | 4.31 | -0.69 | 4.36 | 1.16 |
| **VA15S-L1** | M | 10.8 | 9.48 | -12.22 | 10.72 | -0.74 | 11.49 | 7.18 |
| **VA15S-T11** | S | 11 | 9.42 | -14.67 | 11.12 | 1.09 | 12.04 | 8.27 |
| **AL15L-L5** | S | 7.46 | 6.34 | -15.01 | 7.47 | 0.13 | 8.08 | 8.17 |
| **GA10-T11** | L | 1.87 | 1.68 | -10.16 | 1.87 | 0.00 | 1.82 | -2.67 |
| **MD14-T9** | L | 3.42 | 2.51 | -26.61 | 3.42 | 0.00 | 3.39 | -0.88 |
| **PA15S-T8** | S | 5.33 | 5.79 | 8.63 | 5.35 | 0.38 | 6.67 | 24.67 |
| **MD15A-L1** | S | 5.80 | 4.55 | -21.55 | 5.83 | 0.52 | 5.94 | 1.89 |
| RMSE | |  |  | 0.42 |  | 0.01 |  | 0.32 |

RMSE. Root mean square error (RMSE) =$\sqrt{\frac{\sum\left( Pi-Ei \right)^{2}}{N}}$ with; Pi, Ei: index of the predicted and experimental value in the data set. N: Data set size. BM (Metastatic Bone lesion) =: L: Osteolytic; M: Mixed; S Osteosclerotic.

**Table A.2** Comparison of the measured stiffness values with simulated values predicted at 0.5, 1, and 2 mm mesh element size. Error values were computed with reference to the experimental data.

|  |  | **Measured vs. predicted strengths** | | | | | | |
| --- | --- | --- | --- | --- | --- | --- | --- | --- |
|  |  | **Exp.** | **0.5mm mesh**  **calibration** | | **1mm mesh**  **calibration** | | **2mm mesh**  **calibration** | |
| **Spine ID** | BM | K^Exp^  (kN/mm) | S^hFE^_0.5mm_  (kN/mm) | Error__0.5mm_  (%) | S^hFE^_1mm_  (kN/mm) | Error__1mm_  (%) | S^hFE^_2mm_  (kN/mm) | Error__2mm_  (%) |
| **VA15AL-T11** | S | 19.75 | 22.25 | 12.66 | 18.98 | -3.90 | 23.76 | 20.30 |
| **MD15L-L4** | S | 14.37 | 18.21 | 26.72 | 14.92 | 3.83 | 17.9 | 24.57 |
| **VA15AL-T7** | S | 12.60 | 8.55 | -32.14 | 12.26 | -2.70 | 8.77 | -30.40 |
| **VA15S-L1** | M | 40.83 | 41.05 | 0.54 | 42.16 | 3.26 | 41.89 | 2.60 |
| **VA15S-T11** | S | 36.84 | 36.57 | -0.73 | 36.65 | -0.52 | 38.17 | 3.61 |
| **AL15L-L5** | S | 15.3 | 17.62 | 15.16 | 15.09 | -1.37 | 18.53 | 21.11 |
| **GA10-T11** | L | 9.68 | 9.33 | -3.62 | 9.32 | -3.72 | 8.58 | -11.36 |
| **MD14-T9** | L | 11.82 | 9.01 | -23.77 | 10.7 | -9.48 | 8.65 | -26.82 |
| **PA15S-T8** | S | 9.94 | 18.39 | 85.01 | 9.81 | -1.31 | 18.06 | 81.69 |
| **MD15A-L1** | S | 14.86 | 13.84 | -6.86 | 15.1 | 1.62 | 14.27 | -3.97 |
| RMSE | |  |  | 0.75 |  | 0.23 |  | 1.21 |

RMSE. Root mean square error (RMSE) =$\sqrt{\frac{\sum\left( Pi-Ei \right)^{2}}{N}}$ with Pi, Ei: index of the predicted and experimental value in the data set. Exp.: Experimental data; N: Data set size. BM (Metastatic Bone lesion) =: L: Osteolytic; M: Mixed; S Osteosclerotic.

**Table A.3.** Comparison of measured with the simulated strength values predicted using specimen-specific and “optimized” global calibrated material properties.

|  |  | **Measured vs. predicted strengths** | | | | |
| --- | --- | --- | --- | --- | --- | --- |
|  |  | **Experiment** | **Individual**  **calibration** | | **Global**  **calibration** | |
| **Spine ID** | BM | S^Exp^  (kN) | S^hFE^__I_  (kN) | Error__i_  (%) | S^hFE^ __G_  (kN) | Error__G_  (%) |
| **VA15AL-T11** | S | 8.49 | 8.52 | 0.35 | 7.48 | -11.90 |
| **MD15L-L4** | S | 9.59 | 9.58 | -0.10 | 6.96 | -27.42 |
| **VA15AL-T7** | S | 4.34 | 4.31 | -0.69 | 2.83 | -34.79 |
| **VA15S-L1** | M | 10.8 | 10.72 | -0.74 | 14.3 | 32.41 |
| **VA15S-T11** | S | 11 | 11.12 | 1.09 | 11.4 | 3.64 |
| **AL15L-L5** | S | 7.46 | 7.47 | 0.13 | 5.32 | -28.69 |
| **GA10-T11** | L | 1.87 | 1.87 | 0.00 | 1.99 | 6.42 |
| **MD14-T9** | L | 3.42 | 3.42 | 0.00 | 2.4 | -29.82 |
| **PA15S-T8** | S | 5.33 | 5.35 | 0.38 | 4.98 | -6.57 |
| **MD15A-L1** | S | 5.80 | 5.83 | 0.52 | 4.62 | -20.34 |
| RMSE | |  |  | 0.01 |  | 0.32 |

RMSE. Root mean square error (RMSE) =$\sqrt{\frac{\sum\left( Pi-Ei \right)^{2}}{N}}$ with Pi, Ei: index of the predicted and experimental value in the data set. N: Data set size. BM (Metastatic Bone lesion) =: L: Osteolytic; M: Mixed; S Osteosclerotic.

**Table A.4.** Comparison of measured with the simulated stiffness values predicted using specimen-specific and “optimized” global calibrated material properties.

|  |  | **Measured vs. predicted stiffness** | | | | |
| --- | --- | --- | --- | --- | --- | --- |
|  |  | **Experiment** | **Individual**  **calibration** | | **Global**  **calibration** | |
| **Spine ID** | BM | K^Exp^  (kN/mm) | K^hFE^__I_  (kN/mm) | Error _i_  (%) | K^hFE^__G_  (kN/mm) | Error__G_  (%) |
| **VA15AL-T11** | S | 19.75 | 18.98 | -3.90 | 17.12 | -13.32 |
| **MD15L-L4** | S | 14.37 | 14.92 | 3.83 | 13.66 | -4.94 |
| **VA15AL-T7** | S | 12.60 | 12.26 | -2.70 | 6.67 | -47.06 |
| **VA15S-L1** | M | 40.83 | 42.16 | 3.26 | 29.45 | -27.87 |
| **VA15S-T11** | S | 36.84 | 36.65 | -0.52 | 27.26 | -26.00 |
| **AL15L-L5** | S | 15.3 | 15.09 | -1.37 | 13.83 | -9.61 |
| **GA10-T11** | L | 9.68 | 9.32 | -3.72 | 6.56 | -32.23 |
| **MD14-T9** | L | 11.82 | 10.7 | -9.48 | 6.97 | -41.03 |
| **PA15S-T8** | S | 9.94 | 9.81 | -1.31 | 13.27 | 33.50 |
| **MD15A-L1** | S | 14.86 | 15.1 | 1.62 | 10.77 | -27.52 |
| RMSE | |  |  | 0.23 |  | 0.79 |

RMSE. Root mean square error (RMSE) =$\sqrt{\frac{\sum\left( Pi-Ei \right)^{2}}{N}}$ with Pi, Ei: index of the predicted and experimental value in the data set. N: Data set size. BM (Metastatic Bone lesion) =: L: Osteolytic; M: Mixed; S Osteosclerotic.

**Table A.5** Vertebral-specific rheological model parameters derived from the FE simulation.

| **Spine ID** | **BM** | $\boldsymbol{E}_{\boldsymbol{0}}^{\boldsymbol{0}}$  $\left[ \boldsymbol{GPa} \right]$ | $\boldsymbol{E}_{\boldsymbol{0}}^{\boldsymbol{1}}$  $\left[ \boldsymbol{GPa} \right]$ | $\boldsymbol{E}_{\boldsymbol{0}}^{\boldsymbol{2}}$  $\left[ \boldsymbol{GPa} \right]$ | $\boldsymbol{\eta}_{\boldsymbol{0}}^{\boldsymbol{1}}$  $\left[ \boldsymbol{MPa\cdot s} \right]$ | $\boldsymbol{\eta}_{\boldsymbol{0}}^{\boldsymbol{2}}$  $\left[ \boldsymbol{kPa\cdot s} \right]$ | $\boldsymbol{\sigma}_{\boldsymbol{0}}^{\boldsymbol{-}}$  $\left[ \boldsymbol{MPa} \right]$ | $\boldsymbol{\sigma}_{\boldsymbol{0}}^{\boldsymbol{+}}$  $\left[ \boldsymbol{MPa} \right]$ | $\boldsymbol{\tau}_{\boldsymbol{0}}$  $\left[ \boldsymbol{MPa} \right]$ |
| --- | --- | --- | --- | --- | --- | --- | --- | --- | --- |
| VA15AL-T11 | S | 3.2 | 0.87 | 4.64 | 0.26 | 0.45 | 35.6 | 24.4 | 19.8 |
| MD15L-L4 | S | 3.2 | 0.87 | 4.64 | 0.26 | 0.45 | 48.6 | 33.3 | 27.02 |
| VA15AL-T7 | S | 5.2 | 1.42 | 7.54 | 0.42 | 0.73 | 47.21 | 32.35 | 26.25 |
| VA15S-L1 | M | 4 | 1.09 | 5.8 | 0.33 | 0.56 | 18.6 | 12.75 | 10.34 |
| VA15S-T11 | S | 3.8 | 1.03 | 5.51 | 0.31 | 0.53 | 26.94 | 18.4 | 14.98 |
| AL15L-L5 | S | 3.2 | 0.87 | 4.64 | 0.26 | 0.45 | 49.43 | 33.87 | 27.48 |
| GA10-T11 | L | 4 | 1.09 | 5.8 | 0.33 | 0.56 | 25 | 17.13 | 13.9 |
| MD14-T9 | L | 4.4 | 1.2 | 6.38 | 0.36 | 0.62 | 43.6 | 29.88 | 24.24 |
| PA15S-T8 | S | 2.2 | 0.6 | 3.2 | 0.18 | 0.31 | 38.88 | 26.64 | 21.62 |
| MD15A-L1 | S | 4 | 1.09 | 5.8 | 0.33 | 0.56 | 38.32 | 26.26 | 21.31 |

BM (Metastatic Bone lesion) =: L: Osteolytic; M: Mixed; S Osteosclerotic. Material parameters for a poreless cortical bone: $E_{0}^{1}$: long-term equilibrium elastic modulus; low $\boldsymbol{E}_{\boldsymbol{0}}^{\boldsymbol{0}}$ : and high $E_{0}^{2}$: strain rate viscoelastic modulus. Viscosity parameter: low $\eta_{0}^{1}$: and high $\eta_{0}^{2}$: strain rate and computed bone tissue compressive $\sigma_{0}^{-}$, tensile $\sigma_{0}^{+}$, and $\tau_{0}$: shear stress
