# Supplementary figures and images for: CT-based finite element simulating spatial bone damage accumulation predicts metastatic human vertebrae strength and stiffness
Source: Front Bioeng Biotechnol. 2024 Jul 23;12:1424553. doi: 10.3389/fbioe.2024.1424553 (PMC11300227; doi:10.3389/fbioe.2024.1424553)

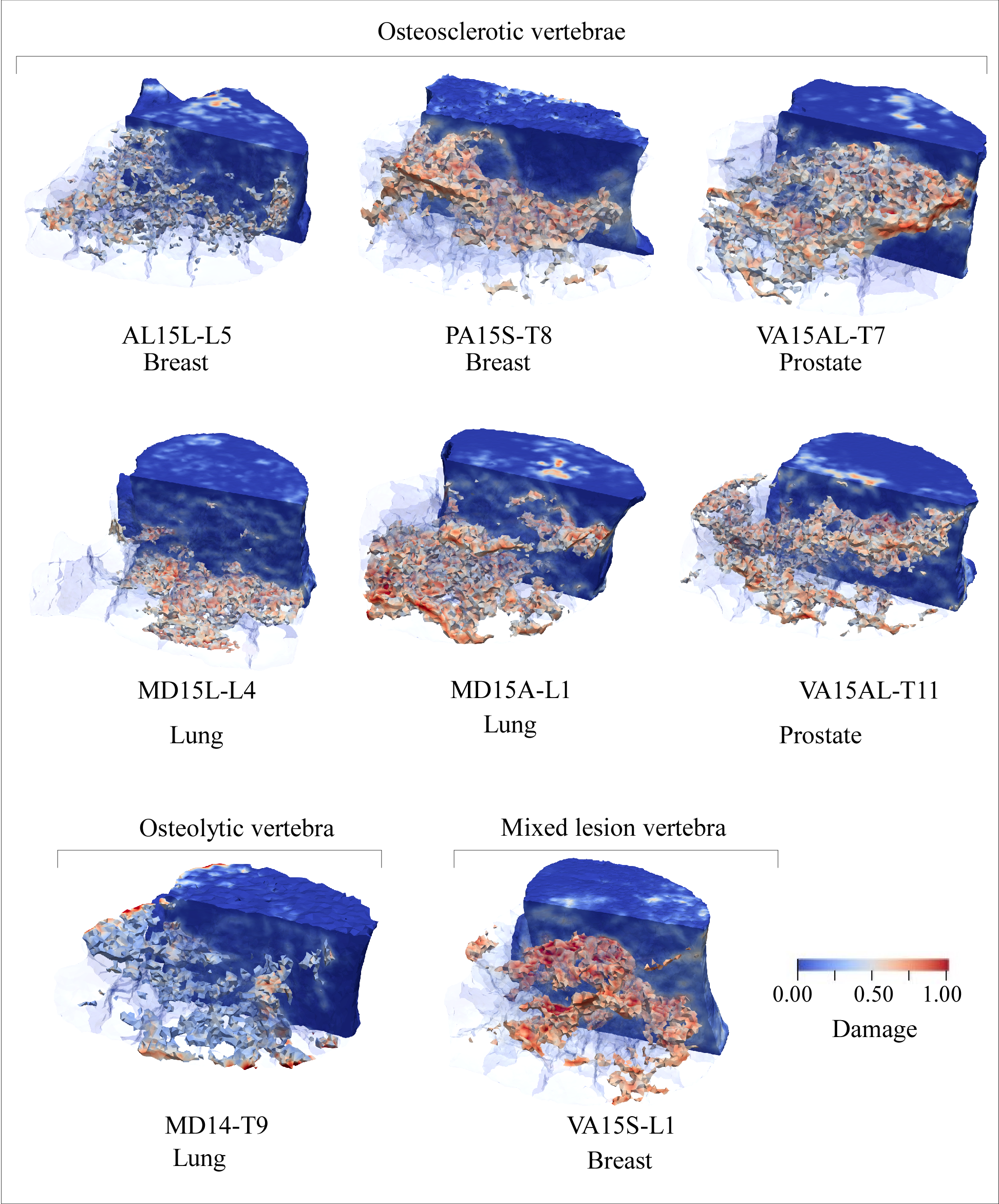

Supplement: Supplementary file 2 [file Image1.TIF]
